# Sphingomonas paucimobilis Septic Shock in an Immunocompetent Patient

**DOI:** 10.7759/cureus.26720

**Published:** 2022-07-10

**Authors:** Bailey Alkhatib, Eric Veytsman, Linda Klumpp, Edwin Hayes

**Affiliations:** 1 Infectious Diseases, University of South Carolina School of Medicine Columbia, Columbia, USA; 2 Infectious Diseases, Prisma Health Richland, Columbia, USA

**Keywords:** antimicrobial resistance, immunocompetent, sphingomonas paucimobilis, septic shock, infectious disease medicine

## Abstract

*Sphingomonas paucimobilis *usually exhibits low virulence likely secondary to its lack of lipopolysaccharide A. Infections caused by *S.* *paucimobilis* more commonly afflict immunocompromised patients. Some case reports document pneumonia, osteomyelitis, pyomyoma, and septic arthritis secondary to* S. paucimobilis *in immunocompetent patients.

*S. paucimobilis* bacteremia is associated with underlying conditions, including malignancy, diabetes mellitus, end-stage renal disease, and chronic obstructive pulmonary disease. Bacteremia has the potential to lead to septic shock. Antimicrobial effectiveness varies, and the mechanism that leads to resistance has not yet been elucidated. This underscores the importance of antimicrobial susceptibility testing.

We present a unique case of community-acquired *S. paucimobilis *bacteremia and resultant septic shock in an immunocompetent patient. A 90-year-old female with a history of chronic kidney disease, acute colonic infarction status post colostomy, gastroesophageal reflux disease, hypertension, supraventricular tachycardia, and schizoaffective disorder presented to the emergency department with hypotension and altered mental status. Urinalysis and chest X-ray were unremarkable. Antibiotic therapy with cefepime was initiated following gram stain, which showed gram-negative rods. The blood culture revealed *S.* *paucimobilis*. The patient was discharged with the plan to enter hospice care.

## Introduction

Nearly 100 species are encompassed within the *Sphingomonas* genus. *Sphingomonas paucimobilis*, previously known as *Pseudomonas* *paucimobilis*, is a strictly aerobic, weakly oxidase-positive, catalase-positive, non-fermenting, gram-negative rod whose colonies grow on blood agar and produce a yellow pigment [1]. *Paucimobilis* is indicative of the organism's limited motility, despite the single polar flagellum. The ideal manner of identification of this bacterium is via matrix-assisted laser desorption ionization time of flight (MALDI-TOF) mass spectrometry [1,2].

*S. paucimobilis* is found in nature in soil and water and has also been isolated from nosocomial settings in water systems and hospital equipment [2]. Due to its ability to form biofilms, it has also been recovered from indwelling devices [3]. Infections with this pathogen tend to be found in immunocompromised patients and rarely in immunocompetent patients [4,5]. Broad-spectrum β-lactam antibiotics, β-lactam-β-lactamase inhibitor combinations, cephalosporins, fluoroquinolones, and carbapenems seem to be the most effective antibiotics [1,2]. We present a unique case of community-acquired *S. paucimobilis* septic shock in an immunocompetent patient with noted ciprofloxacin resistance.

## Case presentation

A 90-year-old female presented to the emergency department from her care facility for evaluation of hypotension. According to the emergency medical services, the patient had weak peripheral pulses and was altered at her residing facility. The patient had a past medical history of chronic kidney disease (CKD), colostomy secondary to ischemia, gastroesophageal reflux disease (GERD), hypertension, supraventricular tachycardia, and schizoaffective disorder. Her vital signs at admission were heart rate of 72 beats per minute, respiratory rate of 15 breaths per minute, blood pressure of 87/54 mmHg, and oxygen saturation of 94% on a 4-liter nasal cannula. Her baseline blood pressure was systolic of 100-110 mmHg and diastolic of 50-60 mmHg. She was disoriented to location, situation, and time. Her colostomy site showed no sign of infection.

Laboratory results on the initial presentation, including a complete blood count (CBC) and comprehensive metabolic panel (CMP), are shown in Table 1. Urinalysis (UA) and chest X-ray (CXR) were unremarkable. An electrocardiogram showed sinus tachycardia with multiple premature complexes and mild diffuse depression. Blood cultures were drawn with concerns over potential sepsis due to her altered mental status and systolic blood pressure of less than 100 mmHg. The patient was appropriately fluid resuscitated, but she remained hypotensive so vasopressors were initiated. She was given 2 g of ceftriaxone based on our hospital's antibiogram for empiric coverage of potential community-acquired pathogens. Although there was no clear source of infection, the leading differential at the time of admission to the internal medicine service was sepsis.

**Table 1:** Initial tabulated CBC and CMP laboratory values CBC: complete blood count; CMP: comprehensive metabolic panel.

Lab	Patient’s values	Normal values
White blood cells	6,600	3.5-10.8 mm^3^
Hemoglobin	12.6	11.0-15.4 g/dL
Platelets	130 x 10^9^	150-400 x 10^9 ^L
Sodium	136	136-145 mmol/L
Potassium	4.6	3.5-5.1 mmol/L
Chloride	99	98-107 mmol/L
Bicarbonate	15	20-30 mmol/L
Anion gap	22	6-16 mmol/L
Blood urea nitrogen	181	7-20 mg/dL
Creatinine	4.29	0.57-1.11 mg/dL
Magnesium	2.3	1.6-2.6 mmol/L
Lactate	1.6	0.5-2.2 mmol/L
Procalcitonin	0.1	<0.10 ng/mL
Troponin	0.03	<0.03 ng/mL

The following day, the blood culture gram stain revealed gram-negative rods. The patient was started on piperacillin-tazobactam with renal adjustment. Piperacillin-tazobactam was promptly discontinued and changed to cefepime according to our hospital's antibiogram for better empiric coverage. The patient’s renal function improved to a blood urea nitrogen (BUN) of 130 mg/dL and creatinine of 2.62 mg/dL. She remained afebrile with no leukocytosis. On day two of admission, the patient’s norepinephrine requirement increased to 18 mcg/minute. Goals of care were discussed with the family members who decided on do not resuscitate/do not intubate and comfort care aligned best with the patient’s wishes. The patient was discharged back to her healthcare facility to eventually be admitted to hospice.

A week following the initial lab draws, the blood culture resulted in *S. paucimobilis*. The organism’s susceptibility to various antibiotics is shown in Table 2. The bacterium was pan susceptible except for intermediate resistance to ciprofloxacin.

**Table 2:** Antibiotic susceptibility of Sphingomonas paucimobilis MIC: minimum inhibitory concentration; S: susceptible; I: intermediate.

Antibiotic	Manual MIC (ug/mL)	Letter designation of resistance level
Amikacin	≤4	S
Cefepime	≤2	S
Ceftazidime	≤2	S
Ceftriaxone	≤1	S
Ciprofloxacin	2	I
Gentamicin	≤1	S
Imipenem	≤4	S
Levofloxacin	≤0.5	S
Meropenem	≤4	S
Piperacillin + tazobactam	≤8	S
Tobramycin	2	S
Trimethoprim + sulfamethoxazole	≤0.5/9.5 u	S

## Discussion

In 1974, the Centers for Disease Control and Prevention (CDC) labeled a novel bacterium CDC group IIk, biotype 1 [1]. Holmes et al. further described this species under the name *Pseudomonas paucimobilis* in 1977 [1]. Throughout the late 1970s, various researchers reported the first clinical presentations of *P.* *paucimobilis*, including meningitis, a lower extremity ulcer, and septicemia [6-8]. Yabuuchi et al. further analyzed the bacterium’s structure, which led to its reclassification as *S. paucimobilis *in 1990 [9]. Since undergoing the nomenclature change, *S.* *paucimobilis* has shown itself as an opportunistic bacterium that rarely leads to infections in immunocompetent individuals. A few case reports show notable presentations in immunocompetent individuals but in the forms of pneumonia, osteomyelitis, septic arthritis, and pyomyoma [10-12].

A 2010 combined case report and literature review of a total of 42 cases of *S. paucimobilis* found that only three patients had septic shock [2]. All possessed immunocompromising risk factors. Two patients had a malignancy, and one had both a burn injury and an intravascular catheter [2]. Other underlying conditions elucidated in the 2010 literature review included diabetes mellitus, chronic obstructive pulmonary disease, end-stage renal disease (ESRD), alcoholism, liver cirrhosis, acquired immunodeficiency syndrome, immunosuppressant use, chronic steroid use, bone marrow transplant, and chemotherapy [2]. The patient we present had a reported history of CKD, but it had not yet progressed to ESRD. She presented with hypotension, altered mental status, and BUN and creatinine levels indicative of acute renal failure. Despite not meeting systemic inflammatory response syndrome (SIRS) criteria, the patient had evidence of acute renal failure and persistent hypotension that was unresponsive to fluid resuscitation and required vasopressors, increasing suspicion of septic shock [13]. However, the patient's presentation of altered mentation and systolic blood pressure of 87 mmHg did fulfill quick Sepsis-related Organ Failure Assessment (qSOFA) criteria, which is associated with an increased risk for in-hospital mortality [13]. Even with the selection of an antibiotic with empiric coverage, her clinical picture failed to improve, and the family decided to pursue hospice care. To our knowledge, this is the first case revealing *S.* *paucimobilis* as a causative organism of septic shock in an immunocompetent patient.

Of note, the research by Lin et al. indicated the most active antimicrobials against *S. paucimobilis* were levofloxacin and ciprofloxacin; whereas, this individual showed only intermediate susceptibility to ciprofloxacin with a minimum inhibitory concentration of 2 ug/mL [2]. To the best of our knowledge, the patient had not had recent antibiotic exposure, which may suggest the bacterium is further mutating. These mutations may be similar to other gram-negative rods, such as *Pseudomonas* *aeruginosa*, in which the bacterium alters the structure of DNA gyrase, the antibiotic’s targeted enzyme.

## Conclusions

The occurrence of infections with *S. paucimobilis* seems to continue to increase. While the majority of literature does not support this bacterium causing infections of significant clinical consequence, this case demonstrates *S. paucimobilis* bacteremia leading to sepsis and progressing to septic shock in an immunocompetent individual. Further awareness of the organism, its clinical presentation, and proper antibiotic treatment can help prevent further complications.
